# Association of acute COVID-19 severity and long COVID fatigue and quality of life: Prospective cohort multicenter observational study

**DOI:** 10.1097/MD.0000000000042891

**Published:** 2025-09-05

**Authors:** Ligia Pires, Ana Marreiros, Cátia Saraiva, Cláudia Reis, Djamila Neves, Cláudia Guerreiro, José Boleo Tomé, Maria Inês Luz, Margarida Isabel Pereira, Ana Sofia Barroso, Jorge Ferreira, Lucía Méndez Gonzalez, Armin Moniri, Marta Drummond, Joana Berger-Estilita

**Affiliations:** a Pulmonology Department, ULS Algarve, Portimão, Portugal; b Pneumology Group, Hospital Particular do Algarve, Alvor, Portugal; c Faculty of Medicine and Biomedical Sciences, Universidade do Algarve, Faro, Portugal; d Statistics Department, Algarve Biomedical Center–Research Institute, Portugal; e Board of Directors, ULS Algarve, Algarve, Portugal; f Psychiatry Department, ULS Algarve, Portimão, Portugal; g Pulmonology Department, ULS Amadora Sintra, Hospital Dr Fernando da Fonseca, Amadora, Portugal; h Pulmonology Sleep Department, ULS de Entre Douro e Vouga, Feira, Portugal; i Department of Otorhinolaryngology, Sahlgrenska University Hospital, University of Gothenburg, Gothenburg, Sweden; j Regenerative Medicine Program, Department of Biomedical Sciences and Medicine, University of Algarve, Faro, Portugal; k Sleep and Non-Invasive Ventilation Department, ULS São João, Porto, Portugal; l Institute for Medical Education, University of Bern, Bern, Switzerland; m RISE-Health, Centre for Health Technology and Services Research, Faculty of Medicine, University of Porto, Porto, Portugal.

**Keywords:** anxiety, depression, fatigue, long COVID-19, post-traumatic stress, quality of life

## Abstract

Long COVID, or post-COVID-19 condition, is characterized by symptoms persisting beyond 12 weeks after severe acute respiratory syndrome coronavirus 2 infection, affecting individuals regardless of acute disease severity. Fatigue – often linked with depression and anxiety – is among its most debilitating manifestations. However, the associations between fatigue subtypes (physical vs mental), mental health symptoms, and acute disease severity on long-term health-related quality of life (HRQoL) remain unclear. This study examines the relationships between long COVID fatigue, depression, anxiety, acute disease severity, and HRQoL in a post-COVID-19 cohort. This prospective observational cohort study was conducted across 5 Portuguese hospitals between November 2020 and June 2022. Adults (≥18 years) with confirmed severe acute respiratory syndrome coronavirus 2 infection ≥6 months prior and fulfilling World Health Organization criteria for long COVID were included. Acute Coronavirus disease 2019 (COVID-19) severity was classified per World Health Organization definitions. The sampling strategy included patients across the severity spectrum. At 3 months postinfection (T1), patients received physician-led clinical assessments. At 6 months (T2), they attended in-person follow-up visits, completing standardized forms and validated questionnaires assessing post-acute sequelae. Fatigue was reported both binarily (yes/no) and via the chalder fatigue scale (11-item version). Anxiety and depression were assessed using the hospital anxiety and depression scale; post-traumatic stress disorder symptoms with the 14-item post-traumatic stress scale; and HRQoL with the EuroQol-5 dimensions. Descriptive statistics, analysis of variance, chi-square, and correlation analyses (Pearson’s or Spearman’s) were used to evaluate associations. Analyses were performed using SPSS (v27; IBM Corp., Amonk). Among 208 patients, fatigue was significantly associated with anxiety and depression (*P* < .001). Physical fatigue correlated more strongly with depression (*r* = 0.65, *P* < .001) and anxiety (*r* = 0.58, *P* < .001) than mental fatigue (*r* = 0.50 and *R* = 0.48, respectively; *P* < .001). Surprisingly, severe acute COVID-19 cases reported lower fatigue (CFQ: 13.3 ± 8.4) than mild (17.7 ± 7.2) or moderate (17.4 ± 8.0) cases (*P* < .005), and higher HRQoL (EuroQol visual analog scale: 74.3 ± 20.3, *P* = .002). Anxiety symptoms were more common in mild cases (*P* < .001); post-traumatic stress disorder symptoms did not differ by severity. Long COVID fatigue – especially physical – is strongly linked to depression and anxiety. Mild/moderate acute COVID-19 cases show greater fatigue and lower HRQoL than severe cases, highlighting the need for tailored long-term care regardless of initial severity.

## 1. Introduction

Coronavirus disease 2019 (COVID-19), caused by the severe acute respiratory syndrome coronavirus 2 (SARS-CoV-2), manifests with a broad spectrum of clinical presentations, ranging from asymptomatic or mild illness to severe, life-threatening conditions.^[1–3]^ The acute phase of the disease typically lasts up to 4 weeks following diagnosis, with symptom resolution varying among individuals.^[4]^ While many patients recover fully within 12 weeks, a subset continues to experience persistent or new symptoms beyond this period, a condition now recognized as long COVID or post-COVID-19 Condition.^[3,5]^

The World Health Organization (WHO) defines long COVID as the persistence or emergence of new symptoms within 3 months of the initial COVID-19 infection, lasting for at least 2 months, and not attributable to an alternative diagnosis.^[6–8]^ This condition can affect individuals regardless of the severity of their acute illness.^[3,8]^ Research indicates that a significant portion of adults who contract SARS-CoV-2 may experience long COVID, with prevalence estimates ranging from 10% to 26%.^[9]^ A study conducted in Scotland investigated the true prevalence of long COVID at 6, 12, and 18 months, revealing crude prevalence rates of 13.8%, 12.8%, and 16.3%, respectively, along with adjusted figures of 6.6%, 6.5%, and 10.4%.^[10,11]^ Consequently, despite the official cessation of the pandemic, our clinical practices remain dedicated to the ongoing assessment and management of patients suffering from long COVID.

Long COVID is highly heterogeneous, often presenting with neuropsychiatric symptoms such as fatigue, depression, anxiety, and cognitive dysfunction.^[3–5,9,12–14]^ Other frequently reported symptoms include dyspnea, palpitations, anorexia, myalgia, and arthralgia.^[12–14]^ Studies indicate that neuropsychiatric symptoms may persist in up to 90% of hospitalized patients and 25% of nonhospitalized individuals 6 months postinfection.^[13]^ Potential mechanisms underlying these manifestations include brain injury, neuronal degeneration, chronic inflammation, viral persistence, and immune dysregulation.^[14,15]^ Additionally, psychological stressors such as disease-related fears, stigma, and social isolation may contribute to the development and persistence of these symptoms.^[16,17]^

Among the most debilitating features of long COVID is fatigue, which can significantly impair daily functioning.^[12]^ Fatigue in this context is defined as a “decline in physical or mental performance due to physiological, psychological, or peripheral factors linked to COVID-19.”^[18,19]^ Studies suggest that fatigue often coexists with depression and anxiety, yet the precise relationship between these symptoms remains unclear.^[20,21]^ Given the profound personal, social, and economic consequences of neuropsychiatric sequelae in long COVID, a deeper understanding of their interconnections is essential.^[19,22]^

This study aims to examine the association between fatigue presented by patients with long COVID and symptoms of depression and anxiety, as well as its impact on quality of life. Our primary hypothesis is that patients with long COVID who report symptoms of depression and/or anxiety will exhibit a higher prevalence and severity of fatigue compared to those without these neuropsychiatric symptoms. Additionally, we assume that higher levels of fatigue, depression, and anxiety will be negatively associated with daily functioning, pain perception, and overall health-related quality of life (HRQoL) among long COVID patients

Our Secondary hypothesis is that there will be differences in long-term levels of fatigue, neuropsychiatric sequelae, and HRQoL between individuals who have experienced mild acute COVID-19 and severe acute COVID-19.

## 2. Methods

### 2.1. Ethics

The study adhered strictly to the guidelines of the Declaration of Helsinki and received ethical approval (Ethical Committee No. 141/21) from the Ethical Committee of Algarve University Hospital Centre, Faro, Portugal, on September 6, 2021. Additionally, the study followed the STROBE guidelines for observational studies.^[23]^

### 2.2. Study design and setting

This study was designed as a prospective observational cohort study, conducted between November 2020 and June 2022, in 5 Portuguese hospitals, including 1 private and 4 public institutions. A full study protocol has been published elsewhere.^[24]^ The study population consisted of symptomatic post-COVID-19 patients who attended specialized post-COVID-19 outpatient clinics for follow-up care. Patients were referred either by their primary healthcare providers or following hospitalization for COVID-19.

### 2.3. Study outcomes

#### 2.3.1. Primary outcome

To determine whether patients with long COVID who experience symptoms of depression and/or anxiety have a higher prevalence of fatigue and report lower HRQoL, compared to those without these neuropsychiatric symptoms.

#### 2.3.2. Secondary outcomes

To assess whether physical fatigue is more strongly associated with depression and anxiety compared to mental fatigue and evaluate whether patients experiencing both depression and anxiety have higher levels of both physical and mental fatigue than those with either condition alone.

To determine whether patients with mild or moderate acute COVID-19 experience higher levels of long-term fatigue than those with severe disease and explore whether the severity of the acute illness influences the persistence and type of fatigue (physical vs mental) in long COVID.

To examine whether individuals with mild acute COVID-19 have a higher prevalence of anxiety symptoms in the long COVID phase compared to those with moderate or severe disease and assess whether depression and post-traumatic stress disorder (PTSD) symptoms differ based on the severity of acute COVID-19.

To evaluate whether patients with mild or moderate acute COVID-19 report lower HRQoL scores in the long COVID phase than those with severe disease and investigate the extent to which fatigue, depression, and anxiety are associated with impairments in daily activities, pain perception, and emotional well-being.

### 2.4. Inclusion and exclusion criteria

Inclusion and exclusion criteria have been described in the study protocol.^[24]^ In summary, Participants were eligible if they were 18 years or older, had a confirmed SARS-CoV-2 infection at least 6 months before enrollment, and experienced persistent symptoms meeting the WHO definition of post-COVID-19 disease. They also needed to have attended a post-COVID-19 follow-up consultation and provided written informed consent. COVID-19 diagnosis was confirmed through reverse transcription polymerase chain reaction or rapid antigen testing within 7 days of symptom onset. Exclusion criteria included preexisting psychiatric disorders, neurological conditions (e.g., stroke, Parkinson’s, Alzheimer’s, amyotrophic lateral sclerosis), and chronic fatigue predating COVID-19. Patients who had undergone invasive mechanical ventilation were excluded to avoid confounding from post-ICU syndrome. Additionally, individuals with cognitive or physical impairments that prevented questionnaire completion were not included.

### 2.5. Data collection

According to protocol,^[24]^ data collection occurred at 2 time points. At T1 (three months postinfection), patients underwent a comprehensive medical assessment conducted by physicians at the post-COVID-19 clinics. This assessment collected detailed demographic data, including educational background, preexisting medical conditions, body mass index, smoking habits, and information regarding acute COVID-19 severity, symptoms, and treatments received. Six months post-COVID-19 diagnosis (T2), patients attended a second in-person follow-up visit at the post-COVID-19 outpatient clinics. During this consultation, they completed standardized clinical research forms and validated questionnaires assessing post-acute sequelae of COVID-19, health status, fatigue, anxiety, depression, and PTSD. These assessments provided a structured evaluation of the long-term impact of COVID-19 on physical and mental health.

#### 2.5.1. Assessment of COVID-19 severity

COVID-19 severity was categorized according to the WHO classification system.^[25]^ Mild disease presented with symptoms but no radiographic pneumonia. Moderate disease included pneumonia on imaging without requiring supplemental oxygen. Severe cases involved pneumonia with respiratory distress, a respiratory rate >30 breaths/min, or oxygen saturation ≤93% on room air. Critical cases required intensive care, including mechanical ventilation, high-flow oxygen, or treatment for septic shock and multi-organ failure.

#### 2.5.2. Assessment of fatigue

Fatigue was initially self-reported in a binary format (yes/no) during clinical evaluations using the post-acute sequelae of COVID-19 Checklist screening questionnaire.^[26]^ We then used the chalder fatigue scale (11-item version) (CFQ-11)^[27]^ to assess fatigue severity over the previous month. The CFQ-11 consists of 11 items rated on a 4-point Likert scale (0–3), with total scores ranging from 0 to 33, where higher scores indicate greater fatigue severity. It includes 2 subdomains: Physical fatigue (7 items; score range: 0–21) and Mental fatigue (4 items; score range: 0–12).

#### 2.5.3. Assessment of depression and anxiety

Symptoms of depression and anxiety were assessed using the hospital anxiety and depression scale (HADS),^[28]^ a validated tool for evaluating psychiatric symptoms in clinical, primary care, and general population settings. The HADS consists of 14 items, with 7 measuring anxiety and 7 measuring depression. Responses were categorized into the following severity ranges: 0 to 7: Normal; 8 to 10: Mild; 11 to 14: Moderate; 15 to 21: Severe. The HADS questionnaire has been validated for use in the Portuguese language, ensuring its applicability to the study population.^[29]^

#### 2.5.4. Assessment of post-traumatic stress disorder (PTSD)

PTSD symptoms were assessed using the post-traumatic stress scale, 14-item version questionnaire,^[30]^ a validated patient-reported outcome measure based on the Diagnostic and Statistical Manual of Mental Disorders, Third Edition, Revised, adapted for post-intensive care syndrome. Patients rated 10 PTSD-related symptoms on a 7-point Likert scale (1 = never to 7 = always), with total scores ranging from 10 to 70. A score above 35 indicated clinically relevant PTSD symptoms. The revised 14-item post-traumatic stress scale, 14-item version, based on Diagnostic and Statistical Manual of Mental Disorders, Fourth Edition criteria, used a cutoff of 45 for probable PTSD.^[31]^ For this study, the questionnaire was modified for post-COVID-19 patients by replacing “intensive care unit (ICU)” with “COVID-19,” while maintaining the original diagnostic cutoffs.

#### 2.5.5. Assessment of health-related quality of life (HRQoL)

We used the EuroQol-5 dimensions (EQ-5D) questionnaire^[32]^ to assess HRQoL. This instrument consists of 2 main components. The first is the EQ-5D-3L Questionnaire, which evaluates 5 key health dimensions: mobility, self-care, usual activities, pain/discomfort, and anxiety/depression. Each domain is rated on a 3-point scale, where 1 indicates no problems, 2 represents moderate problems, and 3 reflects severe problems. These responses calculate a health state index score, ranging from 0 (worst health state) to 1 (best health state).

The second component of the EQ-5D is the EuroQol visual analog scale. This scale allows patients to rate their overall health status from 0 to 100, with 0 representing the worst possible health state and 100 indicating the best possible health state. The EQ-5D has been validated for assessing HRQoL across various disease populations^[33]^ and adapted for Portuguese-speaking populations.^[34]^

### 2.6. Sample size calculation

Fatigue is recognized as one of the most common and persistent symptoms of Long COVID, with previous studies reporting prevalence estimates ranging from 30% to 70% in post-COVID populations.^[13,35–38]^ To ensure a representative sample and allow for meaningful subgroup analysis, we aimed to recruit enough participants across different severity levels of acute COVID-19. We assumed a moderate effect size (Cohen’s *d* = 0.5) for group fatigue differences. Given a statistical power of 80% (β = 0.20) and a significance level of 5% (α = 0.05), a minimum of approximately 200 participants was required to ensure adequate power for the primary analyses.

### 2.7. Statistical analysis

All statistical analyses were performed using SPSS software (version 27; IBM Corp. Amonk). Descriptive statistics were used to summarize the characteristics of the study population. Continuous variables were expressed as mean and standard deviation (SD) if they followed a normal distribution, whereas non-normally distributed variables were presented as median with interquartile range or range. Categorical variables were reported as absolute numbers (n) and percentages (%). To compare groups, we applied different statistical tests depending on the type and distribution of the data. The chi-square (χ²) test was used to examine associations between categorical variables, such as the relationship between COVID-19 severity and fatigue or mental health symptoms. We conducted the Shapiro–Wilk test to determine whether continuous variables followed a normal distribution, which guided the choice of parametric or nonparametric tests for group comparisons. For comparisons of 2 or more independent groups, parametric tests were used when data were normally distributed, such as the analysis of variance, to assess differences in mean fatigue scores across COVID-19 severity groups. If data were non-normally distributed, we applied the Kruskal–Wallis test, a nonparametric alternative suitable for comparing multiple independent groups. To explore the relationship between fatigue and mental health outcomes, we performed linear association analyses using correlation coefficients, Pearson’s or Spearman’s, according to the variables normally distributed. When variables were not normally distributed, we applied Spearman’s rank correlation, which assesses monotonic relationships. Fatigue was analyzed using the total fatigue score (range: 0–33), with subgroup analyses focusing on physical fatigue (range: 0–21) and mental fatigue (range: 0–12). This allowed us to investigate whether different fatigue components were more strongly associated with COVID-19 severity or neuropsychiatric symptoms such as depression, anxiety, and PTSD. A *P*-value < .05 was considered statistically significant for all analyses.

## 3. Results

### 3.1. Patient baseline characteristics

**Figure 1.**
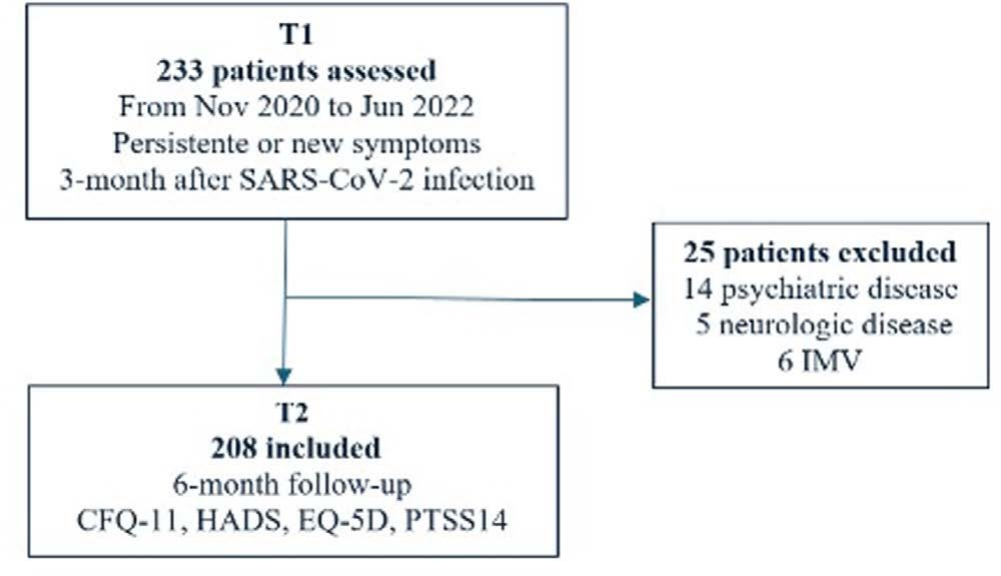
Study flow diagram. CFQ-11 = chalder fatigue scale (11-item version), EQ-5D = EuroQol-5 dimensions, HADS = hospital anxiety and depression scale, IMV = invasive mechanical ventilation, PTSS-14 = post-traumatic stress scale, 14-item version, SARS-CoV-2 = severe acute respiratory syndrome coronavirus 2.

A total of 233 patients with long COVID-19 were initially enrolled in the study, of whom 208 met the inclusion criteria and were analyzed during the study period (Fig. 1). The distribution of participants across study centers was as follows: Alvor Particular Hospital (18.3%, n = 38), Amadora Sintra Hospital (5.3%, n = 11), Faro Hospital (10.1%, n = 19), São Sebastião Hospital (2.4%, n = 5), and Portimão Hospital, which had the most significant proportion of patients (63.9%, n = 133). All 208 participants completed the T2 follow-up questionnaires at a mean of 6.9 months after their initial SARS-CoV-2 positive test.

The median age of the study population was 55.0 years [Q1: 43; Q3: 67.0]. Age distribution varied by disease severity, with mild cases having a median age of 52.0 years [40.0; 61.5], while severe cases had a higher median age of 60.5 years [48.0; 70.0]. Most of the sample was female (53.4%, n = 111), and a significant association was found between female gender and mild acute COVID-19 (*P* < .001). Regarding smoking status, 65.3% (n = 130) of patients were nonsmokers. Patient characteristics according to COVID-19 disease severity are depicted in Table 1.

**Table 1:** Sociodemographic and clinical characteristics of the study population and COVID-19 severity.

		COVID-19 severity			
	Total N = 208	Mild N = 97 (46.6%)	Moderate N = 29 (13.9%)	Severe N = 82 (39.4%)	*P*-value
Age, mean (SD)	55.0 (±14.8)	62.5 (±69.5)	69.1 (±72.8)	59.5 (±13.8)	**<.001**
Median [Q1; Q3]	55.0 [43.0; 68.0]	52.0 [40.0; 62.0]	58.0 [45.0; 70.5]	60.5 [48.0; 70.0]	
Female, N (%)	111 (53.4)	73 (65.8)	15 (13.5)	23 (20.7)	**<.001**
Smoking habits, N (%)					**.024**
Smoker	23 (11.6)	15 (65.2)	5 (21.7)	3 (13.0)	
Former smoker	46 (23.1)	15 (32.6)	7 (15.2)	24 (52.2)	
Nonsmoker	130 (65.3)	63 (48.5)	15 (11.5)	52 (40)	
UPY, mean (SD)	10.3 (± 21.8)	8.2 (± 17.6)	9.6 (±20.9)	12.9 (±26.1)	**<.001**
BMI mean (SD)	29.3 (±5.6)	29.7 (±6.1)	28.2 (±6.2)	29.2 (±5.0)	**.037**
Referral, N (%)		96 (46.4)	29 (14.0)	82 (39.6)	**<.001**
Health workers	25 (12.1)	20 (80.0)	5 (20.0)	0 (0.0)	
Hospital	105 (50.7)	7 (6.7)	18 (17.1)	80 (76.2)	
Primary care	77 (37.2)	69 (89.6)	6 (7.8)	2 (2.6)	
Hospital, N (%)		97 (46.6)	29 (13.9)	82 (39.4)	**<.001**
Alvor	38 (18.3)	31 (81.6)	5 (13.2)	2 (5.3)	
Amadora/Sintra	11 (5.3)	2 (18.2)	3 (27.3)	6 (54.5)	
Faro	21 (10.1)	4 (19.0)	3 (14.3)	14 (66.7)	
Feira	5 (2.4)	3 (60.0)	2 (40.0)	0 (0.0)	
Portimão	133 (63.9)	57 (42.9)	16 (12.0)	60 (45.1)	
Test type					
PCR	193 (92.8)				
TRAG	15 (7.2)				
Arterial hypertension	74 (36.1)	24 (32.4)	10 (13.5)	40 (54.1)	**.004**
Heart disease	21 (10.2)	9 (42.9)	4 (19.0)	8 (38.1)	.783
Diabetes mellitus	25 (12.1)	3 (12.0)	5 (20.0)	17 (68.0)	**<.001**
Chronic kidney disease	4 (1.9)	2 (50.0)	0 (0.0)	2 (50.0)	.704
Respiratory disease	22 (11.8)	10 (45.5)	4 (18.2)	8 (36.4)	.811
Type of respiratory disease					.483
Asthma	15 (71.4)	6 (40.0)	3 (20.0)	6 (40.0)	
COPD	4 (19.0)	2 (50.0)	0 (0.0)	2 (50.0)	
Other	2 (9.5)	2 (100)	0 (0.0)	0 (0.0)	
Immunosuppression	11 (5.3)	5 (45.5)	3 (27.3)	3 (27.3)	.390
Admitted to Hospital	96 (46.2)	2 (2.1)	14 (14.6)	80 (83.3)	**<.001**
Admitted to ICU	27 (13)	0 (0.0)	0 (0.0)	27 (100)	**<.001**
Home isolation, d, mean (SD)	10.2 (±6.8)	10.0 (±5.2)	12.4 (±8.1)	9.6 (±7.8)	**.009**

Bold indicates significant for *P* < .05.

AF = atrial fibrillation, BMI = body mass index, COPD = chronic obstructive pulmonary disease, COVID-19 = Coronavirus disease 2019, HF = heart failure, ICU = intensive care unit, N = number of cases, PCR = polymerase chain reaction, Q1 = first quartile, Q3 = third quartile, SD = standard deviation, TRAG = rapid antigen testing, UPY = pack/year units.

During the acute phase of COVID-19, 46.2% (n = 96) of patients required hospitalization, while 27% (n = 13) required intensive care with oxygen support via high-flow nasal cannula. The presence of arterial hypertension as a preexisting comorbidity was significantly associated with severe COVID-19 (*P* = .004), with 54.1% (n = 40) of hypertensive patients experiencing severe disease. Similarly, diabetes mellitus was strongly associated with COVID-19 severity (*P* < .001), with 68.0% (n = 17) of diabetic patients experiencing severe COVID-19, whereas 51.6% (n = 94) of nondiabetic patients had a mild disease course. No statistically significant associations were observed between COVID-19 severity and the presence of heart disease, chronic kidney disease, preexisting respiratory disease, or immunosuppression.

#### 3.1.1. Primary outcome: association of depression and anxiety in long COVID patients with a prevalence of fatigue

A statistically significant association was observed between fatigue symptoms in long COVID and the presence of neuropsychiatric symptoms, including anxiety, depression, or both (*P* < .001). Patients experiencing depression, anxiety, or both reported significantly higher fatigue levels compared to those without these neuropsychiatric symptoms.

#### 3.1.2. Secondary outcome: correlation between the type of fatigue and depression and/or anxiety symptoms among patients post-COVID-19

The mean total fatigue score, assessed using the CFQ-11 Likert scale, was 15.9 ± 8.1. Notably, significant differences emerged based on the severity of the acute COVID-19 episode; patients who experienced severe acute COVID-19 reported lower fatigue levels (13.3 ± 8.4) compared to those with mild (17.7 ± 7.2) and moderate disease (17.4 ± 8.0), with a statistically significant difference (*P* < .005). Similar patterns were observed in the CFQ physical fatigue subscale, where the overall mean score was 10.4 ± 5.4. Severe COVID-19 patients reported significantly lower physical fatigue scores (8.6 ± 5.5) than patients with moderate (11.0 ± 5.2) or mild disease (11.7 ± 5.0) (*P* = .005). This trend was consistently seen also in the CFQ mental fatigue subscale; severe cases experienced less mental fatigue (4.8 ± 4.3) compared to moderate (6.4 ± 4.0) and mild (6.6 ± 3.2) cases, *P* < .001.

Correlation analysis confirmed a stronger relationship between physical fatigue and neuropsychiatric symptoms, with depression (*r* = 0.65, *P* < .001) and anxiety (*r* = 0.58, *P* < .001) showing higher associations than mental fatigue (*r* = 0.50 for depression, *r* = 0.48 for anxiety, both *P* < .001).

#### 3.1.3. Secondary outcome: correlation between long-COVID fatigue and acute disease severity

Statistically significant differences in fatigue levels were observed among patients based on the severity of their acute COVID-19. Patients who had severe acute COVID-19 reported lower fatigue levels (total CFQ score: 13.3 ± 8.4) compared to those with mild (17.7 ± 7.2) and moderate (17.4 ± 8.0) disease (*P* < .005).

This trend was also evident in the CFQ physical fatigue subscale, where the total sample had an average score of 10.4 ± 5.4. Patients with severe disease had the lowest physical fatigue scores (8.6 ± 5.5), while those with moderate (11.0 ± 5.2) and mild (11.7 ± 5.0) disease reported higher fatigue (*P* = .005).

Similarly, on the CFQ mental fatigue subscale, patients with severe disease reported the lowest scores (4.8 ± 4.3), whereas those with moderate (6.4 ± 4.0) and mild (6.6 ± 3.2) disease experienced higher mental fatigue (*P* < .001).

A statistically significant negative correlation was found between long-COVID fatigue and acute disease severity, meaning that patients with more severe acute COVID-19 experienced lower fatigue levels in the long term. This trend was strongest for total fatigue, with mild and moderate cases showing higher long-term fatigue levels and severe cases exhibiting the strongest negative correlation (*r* = −0.35, *P* < .001). A similar pattern was observed for physical fatigue (*r* = −0.33, *P* < .001) and mental fatigue, though the latter had a slightly weaker correlation (*r* = −0.31, *P* < .001).

#### 3.1.4. Secondary outcome: association between long-COVID mental disorders and acute disease severity

Anxiety symptoms, as assessed by the HADS subscale, were within the normal range across the total study population (mean: 7.8 ± 4.8) and among patients with severe acute COVID-19 (mean: 6.3 ± 4.5). However, a statistically significant difference was observed in patients with mild acute COVID-19, who had a mean anxiety score of 8.9 ± 4.6, reaching the threshold for mild anxiety symptoms (*P* < .001).

In contrast, symptoms of depression and post-traumatic stress (PTSS) remained within the normal range across all severity levels of acute COVID-19, with no significant differences observed (*P* < .001).

#### 3.1.5. Secondary outcome: association of fatigue and mental health disorders with HRQoL after COVID-19

Patients who experienced severe COVID-19 reported a higher overall HRQoL, as measured by the EuroQol visual analog scale, with a mean score of 74.3 (±20.3) compared to 67.6 (±19.0) in mild cases and 64.3 (±19.8) in moderate cases (*P* = .002), Table 2.

**Table 2:** Neuropsychiatric symptoms and COVID-19 severity.

		COVID-19 severity			
	Total N = 208	Mild N = 97 (46.6%)	Moderate N = 29 (13.9%)	Severe N = 82 (39.4%)	*P*-value
CFQ total mean (SD)	15.9 (±8.1)	17.7 (±7.2)	17.4 (±8.0)	13.3 (±8.4)	**.005**
CFQ physical mean (SD)	10.4 (±5.4)	11.7 (±5.0)	11.0 (±5.2)	8.6 (±5.5)	**.004**
CFQ mental mean (SD)	5.6 (±3.8)	6.0 (±3.2)	6.4 (±4.0)	4.8 (±4.3)	**<.001**
HADS anxiety mean (SD)	7.8 (±4.8)	8.9 (±4.6)	8.4 (±5.5)	6.3 (±4.5)	**<.001**
HADS depression mean (SD)	5.7 (±4.0)	6.7 (±3.7)	5.7 (±4.4)	4.5 (±4.0)	**<.001**
PTSS 14 B mean (SD)	39.1 (±22.0)	43.0 (±19.9)	43.8 (±23.8)	33.0 (±22.5)	**<.001**
EQ-VAS mean (SD)	69.8 (± 19.9)	67.6 (±19.0)	64.3 (±19.8)	74.3 (±20.3)	**.002**
EQ mobility N (%)					.359
No problems	135 (70.7)	62 (45.9)	16 (11.9)	57 (42.2)	
Moderate problems	55 (28.8)	22 (40.0)	12 (21.8)	21 (38.2)	
Severe problems	1 (0.5)	1 (100)	0 (0.0)	0 (0.0)	
EQ self-care N (%)					.093
No problems	172 (90.1)	81 (47.1)	23 (13.4)	68 (39.5)	
Moderate problems	18 (9.4)	3 (16.7)	5 (27.8)	10 (55.6)	
Severe problems	1 (0.5)	1 (100)	0 (0.0)	0 (0.0)	
EQ activity N (%)					**.013**
No problems	104 (54.5)	45 (43.3)	8 (7.7)	51 (49.0)	
Moderate problems	82 (42.9)	38 (46.3)	18 (22.0)	26 (31.7)	
Severe problems	5 (2.6)	2 (40.0)	2 (40.0)	1 (20.0)	
EQ pain N (%)					**.026**
No problems	78 (40.8)	27 (34.6)	11 (14.1)	40 (51.3)	
Moderate problems	106 (55.5)	56 (52.8)	17 (16.0)	33 (31.1)	
Severe problems	7 (3.7)	2 (28.6)	0 (0.0)	5 (71.4)	
EQ anxiety N (%)					**.041**
No problems	91 (47.2)	33 (36.3)	12 (13.2)	46 (50.5)	
Moderate problems	82 (42.5)	43 (52.4)	15 (18.3)	24 (29.3)	
Severe problems	20 (10.4)	11 (55.0)	1 (5.0)	8 (40.0)	

CFQ 11 = chalder fatigue scale (11-item version), COVID-19 = Coronavirus disease 2019, EQ-5D = EuroQol 5 dimensions, EQ-VAS = EuroQol visual analog scale, HADS = hospital anxiety and depression scale, N = number of cases, PTSS-14 = post-traumatic stress scale, 14-item version, Q1 = first quartile, Q3 = third quartile, SD = standard deviation.

Regarding specific HRQoL dimensions, a significant association was found between acute COVID-19 severity and difficulties in performing daily activities (*P* = .013), pain (*P* = .026), and anxiety (*P* = .041). However, no statistically significant differences were observed for mobility or self-care. Among individuals without reported activity limitations, 49.0% had experienced severe COVID-19, while 50.5% of those without anxiety symptoms and 51.3% of those without pain had also undergone severe disease. In contrast, most individuals who reported moderate difficulties in these domains had mild acute COVID-19 (46.3% for activity limitations, 52.4% for anxiety, and 52.8% for pain).

There were significant negative correlations between fatigue, mental health symptoms, and HRQoL in post-COVID-19 patients. Total fatigue exhibited the strongest negative correlation with HRQoL (*r* = −0.55, *P* < .001), indicating that higher fatigue levels were linked to a lower perceived quality of life. Both physical fatigue (*r* = −0.52, *P* = .002) and mental fatigue (*r* = −0.50, *P* = .003) also had significant negative associations with HRQoL.

Beyond fatigue, mental health symptoms had a notable impact on HRQoL. Anxiety (*r* = −0.48, *P* = .004) and depression (*r* = −0.51, *P* = .002) were both moderately correlated with poorer quality of life, highlighting their role in diminishing well-being. Post-traumatic stress symptoms (*r* = −0.53, *P* < .001) also showed a strong negative correlation with HRQoL, emphasizing the psychological burden of long COVID.

## 4. Discussion

This study explored the effects of long COVID-19 on fatigue, neuropsychiatric symptoms, and HRQoL in a cohort of 208 patients from multiple healthcare centers. Fatigue emerged as a predominant symptom, with a strong and statistically significant association with anxiety and depression. Patients who initially had a more severe acute COVID-19 infection experienced less long-term fatigue, both physically and mentally, compared to those whose initial COVID-19 illness was mild or moderate. Additionally, fatigue remained prevalent even among patients without neuropsychiatric symptoms, indicating additional contributing factors such as persistent inflammation and autonomic or mitochondrial dysfunctions.

Our findings align with previous studies,^[35–38]^ which report that 20% to 47% of individuals with long COVID experience neuropsychiatric symptoms, consistent with the 28.8% observed in this study. A Spanish study using the HADS and EQ-5D scales found that over 80% of long COVID patients reported fatigue, with 23.5% experiencing depressive symptoms and 35.1% having anxiety.^[37]^ Other exacerbating factors include stigma, negative illness perceptions, and uncertainty about diagnosis and treatment.^[35,36]^

Patients who experienced severe acute COVID-19 reported lower fatigue levels and better overall HRQoL compared to those with mild or moderate disease. This challenges the assumption that greater illness severity leads to more long-term impairment. Our data suggest a somewhat unexpected pattern: individuals who were less severely ill initially reported experiencing higher levels of persistent fatigue months after their infection. This challenges the common assumption that people who have more severe acute illness typically have worse long-term outcomes. Several reasons could explain this counterintuitive finding: on the 1 hand, severe cases might trigger stronger immune or therapeutic responses that could somehow reduce persistent symptoms like fatigue later on, and severely ill patients may have received more intensive medical interventions or rehabilitation support post-hospitalization, reducing fatigue in the long-term.

Additionally, while anxiety levels were lower in the severe COVID-19 group, symptoms of depression and PTSD remained within the normal range across all severity groups. These findings suggest that factors beyond initial disease severity influence post-recovery well-being. Understanding the mechanisms behind these differences could provide valuable insights into long COVID and help develop tailored rehabilitation strategies, particularly for those recovering from mild to moderate cases.

Physical fatigue was more strongly linked to anxiety, while both anxiety and depression were associated with mental fatigue.^[37]^ Fatigue levels varied over time, with physical fatigue being more related to changes in activity patterns.^[39]^ Surveys suggest that physical fatigue is more common than mental fatigue in long COVID,^[9]^ pointing to distinct pathophysiological mechanisms separate from primary psychiatric disorders. A key question is whether fatigue precedes anxiety and depression or vice versa. Some studies suggest that post-viral depression may trigger PTSD symptoms, which, in turn, contribute to fatigue. This cyclical process could lead to a worsening of depression, further fueling fatigue in a self-perpetuating manner.^[40]^ Our findings support the multifactorial nature of long COVID fatigue and highlight the need for a comprehensive treatment approach addressing physical and mental factors.

Further research should explore the timeline of symptom progression – whether fatigue leads to depression and anxiety, or vice versa. Additionally, studies should investigate whether early psychological interventions could prevent or mitigate fatigue in long COVID.

This study has several strengths. The multicenter design, involving 5 hospitals across both public and private sectors, ensures a diverse patient population, increasing the generalizability of the results. Additionally, with data collection at multiple time points (3- and 6-months postinfection), the prospective cohort study design strengthens causality assessment. It minimizes recall bias, providing a more accurate understanding of symptom progression in long COVID.

A key strength of this study is the comprehensive assessment of fatigue and neuropsychiatric symptoms using validated tools. This standardized approach allows for reliable symptom quantification, enabling a thorough evaluation of fatigue, anxiety, depression, and PTSD in long COVID patients. Additionally, the study included patients across all severity levels of acute COVID-19 (mild, moderate, and severe), allowing a comparative analysis of how initial disease severity influences long-term health outcomes. Another strength is the differentiation between physical and mental fatigue, which provides nuanced insights into the nature of post-COVID fatigue. This distinction is valuable for understanding whether different fatigue subtypes have unique underlying mechanisms or clinical implications. The study also highlights sex-based differences, showing a strong association between female gender and increased fatigue, anxiety, and depression, which aligns with emerging evidence on sex-related disparities in long COVID. Moreover, the findings challenge conventional assumptions by demonstrating that mild COVID-19 cases were associated with more significant long-term fatigue and mental health burden than severe cases. This paradox underscores the need for a more refined understanding of long COVID risk factors beyond initial disease severity. By integrating quality of life assessments, the study also provides clinically relevant insights into the broader impact of long COVID, reinforcing the need for targeted follow-up and support strategies.

However, it also has imitations. One major limitation is selection bias, as only patients seeking care at post-COVID-19 clinics were included. This may have led to an overrepresentation of more severe cases while underrepresenting individuals with mild long COVID. Additionally, the exclusion of mechanically ventilated patients limits the generalizability of the findings to the most severe cases. However, this decision was made to avoid including patients with post-ICU admission syndrome, which could introduce other causes of fatigue beyond long COVID. Furthermore, since the study was conducted in 5 Portuguese hospitals, the findings may not fully apply to different populations.

Another key limitation is the lack of a control group, which prevents determining whether the observed symptoms are specific to long COVID or part of a broader post-viral syndrome. The study relied on the CFQ-11 scale to assess fatigue, which is validated for chronic fatigue syndrome but not specifically for long COVID. While long COVID and chronic fatigue syndrome share overlapping clinical and pathophysiological mechanisms, using CFQ-11 alone may limit comparability with studies employing different fatigue assessment tools. This heterogeneity in fatigue measurement methods makes drawing direct comparisons across studies challenging.

Another limitation is the short follow-up period of 6 months, which does not provide insight into the long-term persistence or resolution of symptoms. Additionally, fatigue and neuropsychiatric symptoms can fluctuate over time, but they were only assessed at 2 time points. This limited temporal resolution may have missed essential symptom severity and progression variations.

The broader psychosocial context of the pandemic may have also influenced the results. Factors such as lockdowns, social isolation, and economic stressors could have contributed to the observed mental health symptoms, making it difficult to separate the direct effects of long COVID from pandemic-related distress. Finally, the study relied solely on self-reported questionnaires, introducing subjectivity and potential reporting bias. No objective clinical assessments, such as biomarkers or neurocognitive testing, were conducted to confirm the severity of symptoms. This limitation reduces the ability to validate findings against physiological or laboratory-based measures.

Finally, as this study used bivariate association and correlation analyses without multivariable adjustment for potential covariates, the findings should be interpreted as associations rather than causal relationships. The limited sample size, particularly in some subgroups, restricted our ability to conduct robust multivariable analyses.

In conclusion, this study examined the prevalence and association of fatigue, depression, anxiety, and PTSD in long COVID-19, as well as its impact on quality of life based on disease severity. Mild COVID-19 was more frequent in women and associated with greater fatigue, anxiety, and depression, whereas severe COVID-19 was linked to preexisting diabetes (diabetes mellitus) and hypertension (HTN). Patients with moderate acute illness experienced higher fatigue. However, acute disease severity did not impact PTSD symptoms. Patients with mild or moderate COVID-19 had a lower quality of life, particularly in activity, pain, and anxiety domains, compared to those with severe illness. The findings emphasize the need for ongoing follow-up and support for long COVID patients, even those with mild acute illness, due to its lasting impact on mental health and daily functioning. Future research should explore the role of pharmacological treatments, such as corticosteroids, in neuropsychiatric symptoms.

## Author contributions

**Conceptualization:** Ligia Pires, Cláudia Reis, Armin Moniri, Marta Drummond.

**Data curation:** Ligia Pires, Cátia Saraiva, Cláudia Reis, Djamila Neves, Cláudia Guerreiro, José Boleo Tomé, Maria Inês Luz, Margarida Isabel Pereira, Ana Sofia Barroso, Jorge Ferreira, Lucía Méndez Gonzalez, Joana Berger-Estilita.

**Formal analysis:** Ligia Pires, Marta Drummond.

**Investigation:** Ligia Pires, Ana Marreiros, Cátia Saraiva, Cláudia Reis, Djamila Neves, Cláudia Guerreiro, José Boleo Tomé, Maria Inês Luz, Margarida Isabel Pereira, Ana Sofia Barroso, Jorge Ferreira, Lucía Méndez Gonzalez, Armin Moniri, Marta Drummond, Joana Berger-Estilita.

**Methodology:** Ligia Pires, Ana Marreiros, Cláudia Reis, Ana Sofia Barroso, Jorge Ferreira, Armin Moniri, Marta Drummond.

**Project administration:** Ligia Pires, Cátia Saraiva, José Boleo Tomé, Maria Inês Luz, Margarida Isabel Pereira, Armin Moniri, Marta Drummond.

**Resources:** Ligia Pires, Lucía Méndez Gonzalez.

**Software:** Ligia Pires, Ana Marreiros.

**Supervision:** Ligia Pires, Marta Drummond.

**Visualization:** Ligia Pires, Marta Drummond.

**Writing – original draft:** Ligia Pires, Ana Marreiros, Marta Drummond, Joana Berger-Estilita.

**Writing – review & editing:** Ligia Pires, Ana Marreiros, Marta Drummond, Joana Berger-Estilita.
